# Flexural and Cell Adhesion Characteristic of Phakic Implantable Lenses

**DOI:** 10.3390/medicina59071282

**Published:** 2023-07-10

**Authors:** Kazuo Ichikawa, Kei Ichikawa, Naoki Yamamoto, Rie Horai

**Affiliations:** 1 Chukyo Eye Clinic, Nagoya 456-0032, Aichi, Japan; kei@chukyogroup.jp (K.I.);; 2 General Aoyama Hospital, Toyokawa 441-0103, Aichi, Japan; 3 Support Office for Bioresource Research, Translational Research Headquarters, Fujita Health University, Toyoake 470-1192, Aichi, Japan; naokiy@fujita-hu.ac.jp

**Keywords:** hole-implantable collamer lens (H-ICL), implantable phakic contact lens (IPCL), intraocular lens (IOL), compression load, lens flexion, static Young’s modulus, cell adhesion, immortalized human lens epithelial cells (iHLEC-NY2)

## Abstract

*Background and Objectives*: In this study, we aimed to compare the physical properties of hole-implantable collamer lenses (H-ICLs) and implantable phakic contact lenses (IPCLs) and investigate their flexural and cell adhesion characteristics. *Materials and Methods*: Transverse compression load to achieve lens flexion and static Young’s modulus were measured in H-ICLs and IPCLs using designated equipment. Load was measured both with and without restraining the optic section of the lenses. Adhesion of iHLEC-NY2 cells to the lens surfaces was examined using phase-contrast microscopy, and cell proliferation activity was evaluated using WST-8 assay. *Results*: The H-ICL showed a greater tendency for transverse compression load compared to IPCL, while the IPCL showed a higher Young’s modulus with respect to the force exerted on the center of the anterior surface of the optic section. The joint between the optic section and haptic support in the IPCL was found to mitigate the effects of transverse compression load. Both lens types showed minimal cell adhesion. *Conclusions*: Our findings indicate that H-ICLs and IPCLs exhibit distinct physical properties and adhesive characteristics. The IPCL demonstrated higher Young’s modulus and unique structural features, while the H-ICL required greater transverse compression load to achieve the flexion required to tuck the haptic supports into place behind the iris to fix the lens. The observed cell non-adhesive properties for both lens types are promising in terms of reducing complications related to cell adhesion. However, further investigation and long-term observation of IPCL are warranted to assess its stability and potential impact on the iris. These findings contribute to a better understanding of the performance and potential applications of H-ICLs and IPCLs in ophthalmology.

## 1. Introduction

Laser corneal refractive surgery (e.g., laser in situ keratomileusis (LASIK)) is a quick and easy procedure with lower risk of complications than corneal transplant surgery in patients who meet surgical criteria. Other superficial corneal surgical interventions, such as photorefractive keratectomy (PRK) [1,2,3] and small-incision lenticule extraction (SMILE), have been used to correct high-refractive myopia [4,5]. However, LASIK is contraindicated in patients at risk of ocular surface complications such as dry eye, optic neuropathy, very severe high myopia [6], and those with thin corneas [7,8,9,10,11].

The phakic implantable contact lens (phakic-ICL) is inserted into the eye to correct refractive errors without removal of the natural crystalline lens. Unlike LASIK, phakic-ICL insertion is a reversible procedure wherein the lens can be replaced as needed. It may be used to treat cases with abnormal corneal shape, thickness, or severe myopia. The phakic-ICL is inserted behind the iris in the eye in the space between the iris and lens, which is called the posterior chamber. The development of phakic-ICL insertion surgery dates back to the 1980s, before LASIK was first reported in 1990 [12,13].

Early models, including angle-supported and iris-fixated lenses [14], were phased out due to associated complications such as corneal endothelial decompensation, uveitis, glaucoma, and progressive iris atrophy [15]. Therefore, early phakic-ICL were developed that increased the distance between the lens and the corneal endothelium. In particular, phakic-ICL made from a flexible biocompatible material called collamer (a copolymer of Hydroxyethylmethacrylate (HEMA) and collagen) were developed, which were thought to cause less irritation to the iris and other intraocular tissue and less damage to the corneal endothelium [16]. However, during lens development, there were concerns that insertion into the posterior chamber may impede aqueous humor circulation and increase intraocular pressure, necessitating a peripheral iridotomy procedure to make a pathway for aqueous flow. Improvements were made, and the EVO/EVO+ VISIAN^®®^ Implantable Collamer^®®^ Lens (VISIAN^®®^ Hole-Implantable Collamer Lens: H-ICL) was developed, which has a 0.36 mm diameter hole in the optic center allowing passage of aqueous humor, rendering peripheral iridectomy no longer necessary [17,18,19,20,21].

In contrast, acrylic materials have long been used to make intraocular lenses (IOL) for implantation in aphakic eyes after cataract surgery and have high safety and effectiveness in terms of biocompatibility [22], but they tended to lack flexibility. However, positive results of H-ICL have led to development of a new hydrophilic hybrid acrylic material with a higher water content. Now, several types of Implantable Phakic Contact Lens (IPCL) are available, incorporating features of good lens shape and hole(s) to channel aqueous humor.

A prospective 12-month follow-up study of H-ICL and IPCL reported no statistically significant difference between the two types of lenses in mean postoperative spherical equivalent, corrected visual acuity (VA), or mean decrease in corneal endothelial cell count [23]. However, long-term follow-up is deemed necessary to investigate the incidence of posterior capsule opacification. Furthermore, although different from the material used in IPCL, hydrophilic acrylic IOL have been associated with lens opacification [24].

**Figure 1:**
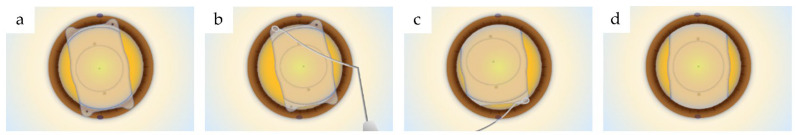
The phakic-ICL insertion: (**a**) insert the folded lens, open it in position between the cornea and iris; (**b**) the lens is secured by haptic supports; (**c**) tuck the haptic supports into the space between the iris and lens (ciliary sulcus); (**d**) lens insertion is complete.

During refractive correction surgery, some sections of the phakic-ICL called haptic plates are tucked into place to position and support the lens behind the iris after intraocular insertion (Figure 1).

The procedure of flexing and tucking the haptics behind the iris is similar to the compressive load measurement procedure for mechanical properties described in ISO 11979-3:2012 (Ophthalmic implants—Intraocular lenses—Part 3: Mechanical properties and test methods) [25]. In simple terms, forces acting on the phakic-ICL when bending and inserting the haptic flaps are attributed to lateral compression (compressive load) from the sides and the restoring forces at the surface of the optic (Young’s modulus). Compressive load is external force applied to the surface of an object when pushed or pressed. Young’s modulus (longitudinal elasticity modulus) is the proportionality constant between strain and stress in the coaxial direction within the elastic range where Hooke’s law holds. It describes the property of a material to return it to its original shape when the applied external force is removed. If the haptics are rigid during this operation, there is a risk of contact with the crystalline lens, which may in turn increase the risk of cataract development.

H-ICL has a clinical history of almost 30 years but that of IPCL is less [15]. Clarification of the differences between H-ICL and IPCL lenses could inform guidelines for future observations. To compare physical properties of H-ICL and IPCL, we: (1) measured compressive load as mechanical property; (2) determined Young’s modulus to find the ability of the material to return to its original shape after an applied external force is removed; and, since cell adhesion varies with lens material and surface treatment; (3) conducted comparative experiments on cell adhesion to H-ICL and IPCL made of different materials. This is the first study to investigate and report these parameters.

## 2. Materials and Methods

### 2.1. Materials

**Figure 2:**
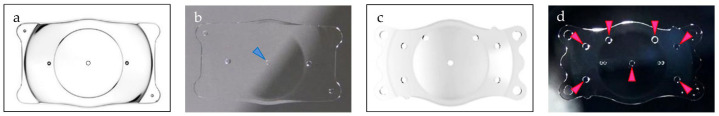
Two types of phakic-ICL: (**a**) outline of H-ICL type; (**b**) photograph of H-ICL. The blue arrowhead indicates a 0.36 mm diameter hole for passage of aqueous humor; (**c**) outline of IPCL type; (**d**) photograph of IPCL. Red arrowheads indicate holes for aqueous humor.

Two types of phakic-ICL were used in the experiments: three H-ICL (EVO/EVO+ VISIAN^®^ Implantable Collamer^®^ Lens, STAAR Surgical Co., Lake Forest, CA, USA) lenses of different lengths, 12.1, 12.6, and 13.2 mm, with the same diopter power −7.00 D; and three IPCL (Implantable Phakic Contact Lens V2.0, Care Group Sight Solution LLP, Vadodara, Gujarat, India) lenses of the same length, 12.5 mm, but different power levels −5.00 D, −8.00 D, and −13.5 D. Two of each of these types (12 lenses) were investigated (Figure 2).

As a control lens in cell adhesion experiments, an IOL with a soft acrylic optic section commonly used in cataract procedures was utilized (iSert^®^ Micro Toric aspheric 1-piece model 355T5, HOYA Corp., Tokyo, Japan).

### 2.2. Measurement of Compressive Load

**Figure 3:**
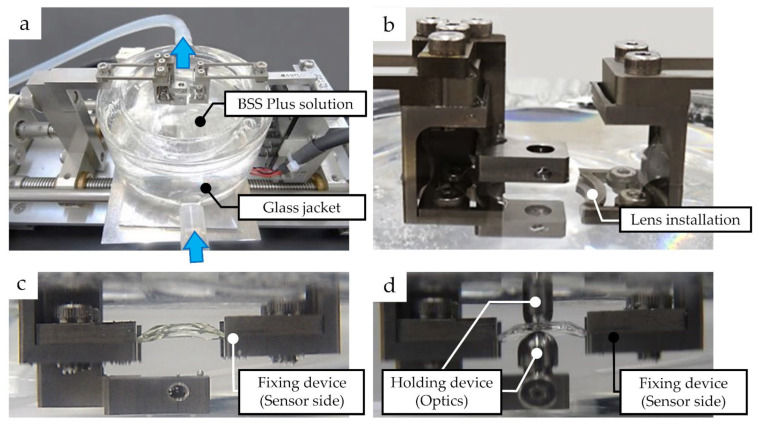
Compression load measurement apparatus: (**a**) BSS Plus solution inside the glass jacket was maintained at 35.0 °C by circulation of heated water (blue arrow); (**b**) installation of the haptic support of H-ICL and IPCL; (**c**) measurement of compression load without the optic section restrained; (**d**) measurement of compression load with the optic section restrained.

The inside of a “glass jacket” was filled with BSS Plus sterile intraocular irrigating solution (BSS Plus solution, Alcon, Inc., Geneva, Switzerland), commonly used during intraocular surgical procedures. This solution was in direct contact with the lens. The solution temperature was maintained at 35.0 °C via a tube circulating water through the solution attached to an Ecoline RE104 Recirculating Chiller (LAUDA-Brinkmann, LP., Marlton, NJ, USA) outside the glass jacket. Computer simulations have reported that human crystalline lens temperatures range from 35.0 °C to 37.5 °C [26]. A dedicated fixture was used to hold the H-ICL and IPCL in place during experiments with multi-purpose material testing apparatus for the intraocular lenses (Imoto Machinery Co., Ltd., Kyoto, Japan). The measurements were taken under two conditions, with and without the optic section restrained (Figure 3).

### 2.3. Measuring Static Young’s Modulus

**Figure 4:**
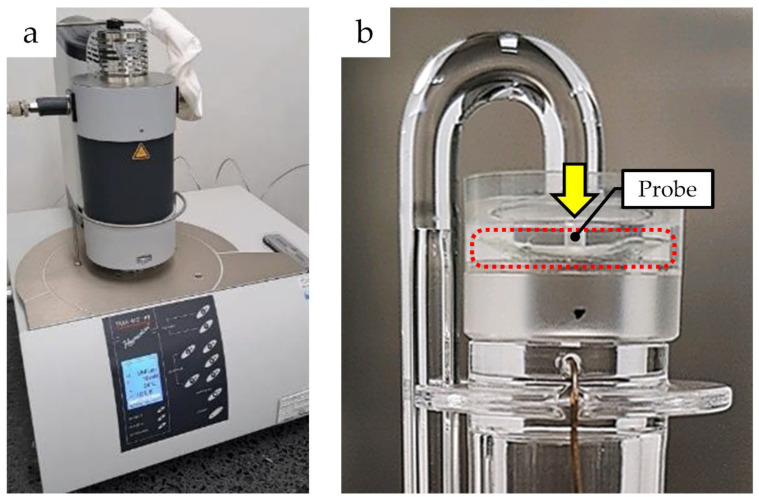
Static Young’s modulus measurement apparatus: (**a**) external appearance of the apparatus; (**b**) the lens is placed in the red dashed line area and a force of 0.1 N is applied.

Similar to compression testing, static Young’s moduli of H-ICLs and IPCLs were measured in the lenses immersed in the solution at 35.0 °C. The measurements were performed with a thermomechanical analyzer (TMA 402 F1 Hyperion^®^, NETZSCH Japan K.K., Kanagawa, Japan) (Figure 4). A force of 0.1 N, was applied by a 1 mm diameter probe to the top surface at the center of the optic section for 5 min then removed. Static Young’s modulus was calculated from the displacement before and that after the application of force.

### 2.4. Cell Adhesion Experiments

In cell adhesion experiments, immortalized human lens epithelial cells (iHLEC-NY2) were used [27]. They were seeded and cultured on H-ICL, IPCL, and control IOL well plates (Costar^®®^ 24-well clear TC-treated multiple-well plate P/N: 3524, Corning Inc., Corning, NY, USA), and adjusted to a cell density of 1 × 10^4^/100 µL/lens or well. Cell growth was observed and recorded using an inverted microscope (Power IX71, Olympus Corp., Tokyo, Japan) and digital camera system (DP-51, Olympus Corp.). Cell proliferation was measured using cell counting kit-8 (Dojindo Laboratories Co., Ltd., Kumamoto, Japan) containing 2-(2-methoxy-4-nitrophenyl)-3-(4-nitrophenyl)-5-(2,4-disulfophenyl)-2H-tetrazolium, monosodium salt (WST-8). Cultures were performed in triplicate for each sample, and cell adhesion and proliferation were evaluated after 1, 4, and 7 days of culture. Cell counting kit-8 reagent diluted in 1/10 volume of culture medium was added to the wells and the reaction proceeded for 3 h at 37.0 °C in a 5% CO_2_ incubator. The supernatant of the culture medium was then transferred to a 96-well clear TC-treated multiple well plate (P/N: 3599, Corning Inc.) and absorbance at 450 nm was measured using a microplate reader (Benchmark Model 550, Bio-Rad Lab., Inc., Hercules, CA, USA).

### 2.5. Statistical Analysis

Data, presented as mean ± standard deviation (SD), were analyzed by Kruskal–Wallis followed by Scheffe’s post hoc test to compare three or more groups of independent data, and Mann–Whitney U was used to compare pairs of groups, using SPSS Statistics 24 (IBM Corporation, New York, NY, USA).

## 3. Results

### 3.1. Compression Load and Comparisons among Lenses

**Figure 5:**
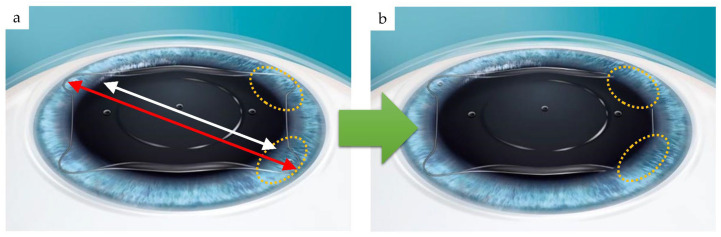
Basis of haptic compression: (**a**) illustration of H-ICL inserted between the cornea and iris. Lens overall length (red line), pupil diameter (white line); (**b**) illustration after inserting the haptic supports in two locations between the iris and crystalline lens (sulcus). The haptic supports are flexed to be inserted in the ciliary sulcus behind the iris.

Compression load is the force acting in the direction of compression when load is applied to the surface of an object. To deform an object, the load required indicates its resistance (hardness). First, the load during transverse compression was measured. The overall length of the IPCL was 12.5 mm, and that of the intermediate size H-ICL was 12.6 mm. To simulate the situation wherein the center of the optic section would align with the center of a dilated pupil of 8 mm diameter to make sure the lens was positioned correctly, we set the overall length of each lens to be 12.5 mm, measured from corner to corner of the haptics, as shown (Figure 5a). To insert the lens into the posterior chamber behind the iris, one must flex the haptics of the lens. Assuming a pupil diameter of 8 mm, one needs to flex the lens by 4.5 mm. Since each haptic is flexed separately, one would flex the lens by half of that amount, which is 2.25 mm. Thus, to measure compression load during this flexion process, the results of experiments to compress the lens by 2.25 mm and 4.5 mm revealed the forces involved in flexion of the lenses (Figure 5).

#### 3.1.1. Optic Section Unrestrained

Table 1 shows the compression load by flexion for H-ICL- and IPCL-type lenses without the optic section restrained.

**Figure 6:**
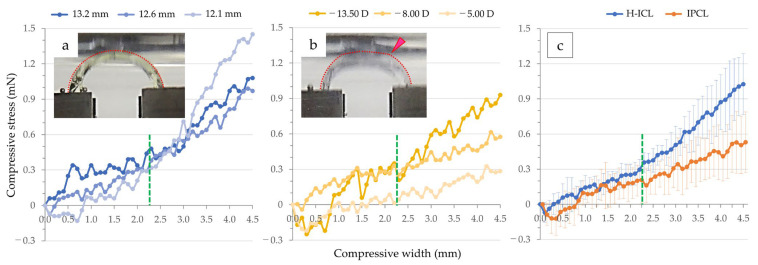
Measurement of compressive load without the optic section restrained: (**a**) measurement results of H-ICL. The entire lens curved like an arch (red dotted line); (**b**) measurement results of IPCL. The junction between the optic section and haptic support became convex (red arrowhead) while bending (red dotted line); (**c**) measurement results of the H-ICL group and the IPCL group. The horizontal axis represents the compressive width (flexion), and the vertical axis represents the compression load. The green dotted line indicates a flexion of 2.25 mm, where one side of the lens haptic support is flexed.

To flex H-ICL by 4.5 mm, the required compression load was greatest for the 12.1 mm length H-ICL, with little difference between the 12.6 mm and 13.2 mm varieties. To achieve 2.25 mm flexion, no difference in compression load was observed among the three H-ICLs (Figure 6a).

To flex the IPCL by 4.5 mm, the variety with a power of −13.50 D required the greatest load, followed by −8.00 D then −5.00 D, indicating an association between lens power and load. To achieve 2.25 mm flexion, the loads required for the −13.50 D and −8.00 D varieties were similar, while −5.00 D required an almost unnoticeable load (Figure 6b). Despite variability in load by lens size and power, grouping them by H-ICL and IPCL type, the H-ICL group required greater loads to achieve 2.25 mm and 4.5 mm flexion than did the IPCL group, albeit not significantly. With the optic section unrestrained, compression load tended to increase with amount of flexion (Figure 6c).

#### 3.1.2. Optic Section Restrained

Table 2 shows the compression load by flexion for H-ICL- and IPCL-type lenses with the optic section restrained.

**Figure 7:**
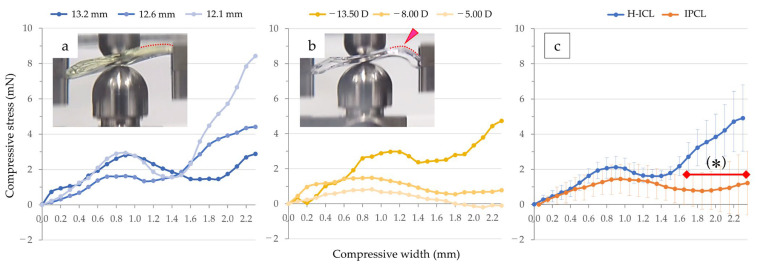
Measurement of compression load with the optic section restrained: (**a**) Measurement results of H-ICL. Up to flexion of 0.9 mm, the H-ICL exhibited a curved shape throughout the lens (red dotted line); (**b**) Measurement results of IPCL. The junction between the optic section and haptic support became convex (red arrowhead) while bending (red dotted line); (**c**) Measurement results of the H-ICL group and IPCL group. From flexion of 1.7 mm to 2.25 mm, the average compression load of H-ICL was significantly higher than that of IPCL (* *p* < 0.05). The horizontal axis represents the compressive width (flexion), and the vertical axis represents the compression load.

To flex H-ICL by 4.5 mm, the required compression load was greatest for the 12.1 mm length H-ICL, and lower for the 12.6 mm then 13.2 mm varieties. By restraining the optic section, the relationship between load and flexion at the lens support in the apparatus became more apparent. By fixing the optic section, the shape of the graph of load against flexion resembled a four-parameter logistic regression curve (cubic function curve). The required load increased for all three varieties up to flexion of 0.8 to 0.9 mm to reach a peak. Thereafter, the required load decreased as flexion increased, and then increased again in proportion to the amount of flexion (Figure 7a). As seen in the video (Video S1), when flexion exceeded 0.8 to 0.9 mm, the area around the junction of the lens optic section and the support component section bent resulting in a temporary decrease in compression load. However, when the area bent sufficiently, the load increased again, primarily due to the optic section exerting pressure.

To flex IPCL by 2.25 mm, the required compression load was greatest for the −13.50 D version and lower for the −8.00 D then −5.00 D varieties. The −13.50 D lens reached a peak load at a slightly larger flexion of approximately 1.1 mm. For the −8.00 D and −5.00 D lenses, similar to the H-ICL, they showed a gradual increase in load until flexion of about 0.8 to 0.9 mm. After that point, load decreased for the −13.50 D lens as flexion increased, and then it increased proportionally to flexion thereafter. As seen in the video (Video S2), compression load decreased as flexion increased in the −8.00 D and −5.00 D lenses and showed no clear increase in load until flexion reached 2.25 mm (Figure 7b).

For both types of lenses, under flexion of approximately 1 mm, where the junction of the optic section and support component section bent, the compression load was about 3 mN for H-ICL varieties 13.2 mm and 12.1 mm, IPCL version −13.50 D, and about 1.5 mN for 12.6 mm H-ICL and −8.00 D IPCL. Compared to when unrestrained, when the optic section was restrained, mean compression load increased 16.4-fold in the H-ICL group and 5.8-fold in the IPCL group to achieve flexion of 2.25 mm being significantly higher in the former (*p* < 0.05) (Figure 7c).

### 3.2. Comparison of Static Young’s Modulus

**Figure 8:**
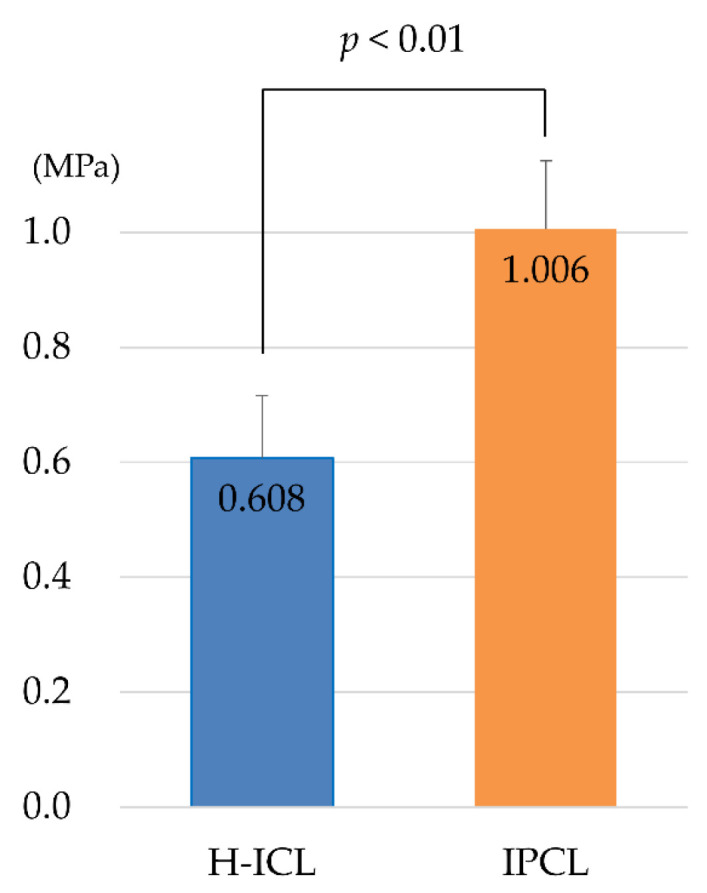
Measurement results of static Young’s modulus. The static Young’s modulus of IPCL was significantly higher than that of H-ICL (*p* < 0.01).

Static Young’s modulus was measured to determine flexion of the lens surface. A higher value indicates a lens is less prone to flexion. By length of H-ICL, Young’s modulus was 0.68 MPa for 12.6 mm, 0.55 MPa for 13.2 mm, and 0.44 MPa for 12.1 mm. There was no significant difference among lens length varieties in the H-ICL group or among power varieties in the IPCL group. However, the mean ± SD Young’s modulus for the H-ICL group was 0.608 ± 0.108 MPa, while that for the IPCL group was 1.006 ± 0.119 MPa, indicating IPCL lenses were less prone to deformation (*p* < 0.01) (Figure 8).

### 3.3. Cell Adhesion to Lens

iHLEC-NY2 cells were observed under an inverted microscope and photographed with a digital camera immediately after seeding and at 1, 4, and 7 days of culture. In conjunction, 200 µL supernatant of the medium reacted with WST-8 reagent for 3 h was transferred to 96-well microplates and absorbance was measured.

#### 3.3.1. iHLEC-NY2 Cell Adhesion Status

**Figure 9:**
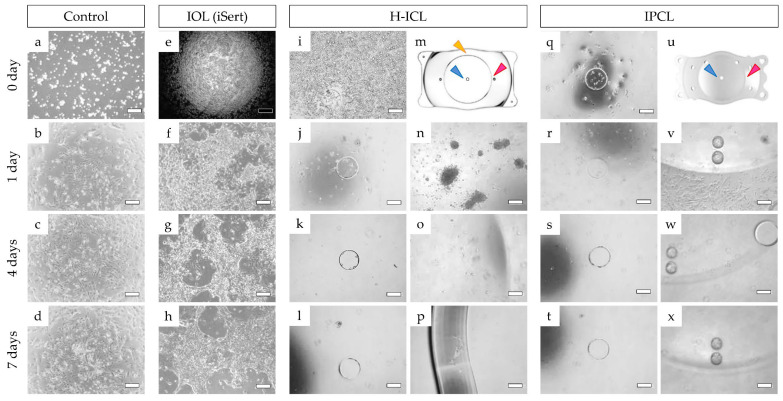
Adhesion of iHLEC-NY2 cells observed using a phase-contrast microscope: (**a**–**d**) adhesion of cells on a multi-well cell culture plate as a control; (**e**–**h**) adhesion of cells on an IOL surface as a control; (**i**–**l**) adhesion of cells near the center of the optic section of H-ICL (blue arrowhead, Figure 9m); (**m**) schematic illustration of the imaging location of H-ICL; (**n**,**o**) adhesion of cells around the periphery of the optic section of H-ICL (red arrowhead, Figure 9m); (**p**) occasional observation of cells on the optical section extending from the optic section to the lens edge of H-ICL (yellow arrowhead, Figure 9m); (**q**–**t**) adhesion of cells near the center of the optic section of IPCL (blue arrowhead, Figure 9u); (**u**) schematic illustration of the imaging location of IPCL; (**v**–**x**) adhesion of cells around the periphery of the optic section of IPCL (red arrowhead, Figure 9u). The scale bar denotes 500 µm.

On IOL and H-ICL, immediately after seeding iHLEC-NY2 cells they remained at the center of the optic section (Figure 9e,i). However, on IPCL, cells were displaced from the center to the peripheral region (Figure 9q). On the control multi-well cell culture plate, the cells were evenly distributed (Figure 9a).

On day 1 of culture, some iHLEC-NY2 cells adhered to the center of the optic section of the H-ICL, but their morphology appeared elongated and spindle-shaped (Figure 9j). Cell clusters were observed in the peripheral region of the H-ICL, but few cells adhered to the H-ICL surface (Figure 9n). In the case of the IPCL, almost no iHLEC-NY2 cells were observed to adhere to the center of the optic section (Figure 9r), but spindle-shaped cells adhered to the surface beyond the periphery of the optical part (Figure 9v). A monolayer of cells was observed to adhere to the surface at the center of the optic section of the IOL (Figure 9f).

On day 4 of culture, a few iHLEC-NY2 cells remained by the sides of the hole in the center of the H-ICL lens, but few cells were observed adhering to the surface of the optic section itself (Figure 9k). In the peripheral region of the optic section, the clusters of iHLEC-NY2 cells observed on day 1 had disappeared, and only a few were observed adhering to the surface (Figure 9o). In the case of IPCL, hardly any iHLEC-NY2 cells were observed adhering to the surface at the center of the optic section (Figure 9s). The cells that were previously observed adhering to the lens beyond the peripheral region of the optic section were also no longer observed (Figure 9w), while cell adhesion was observed on the surface, clustering of iHLEC-NY2 cells was also observed (Figure 9g).

On day 7 of culture, almost no iHLEC-NY2 cells were observed in the center of the optic section of H-ICL (Figure 9l), and only a few scattered iHLEC-NY2 cells were observed adhering to the peripheral region of the optic section (Figure 9p). Almost no iHLEC-NY2 cells were observed in the center of the optic section of IPCL (Figure 9t), and also, almost none were observed adhering to the peripheral region of the optic section (Figure 9x). As for the IOL, iHLEC-NY2 cells were observed adhering to the surface in clustered and stratified layers (Figure 9h). In the control multi-well cell culture plate, iHLEC-NY2 cells grew well and proliferated from day 1 to day 4 (Figure 9b,c) and by day 7 they had almost reached confluence, which is a continuous and densely packed layer filling the available space (Figure 9d).

#### 3.3.2. iHLEC-NY2 Proliferation Activity

Table 3 shows the absorbance values of the control, IOL, H-ICL, and IPCL on culture days 1, 4, and 7.

**Figure 10:**
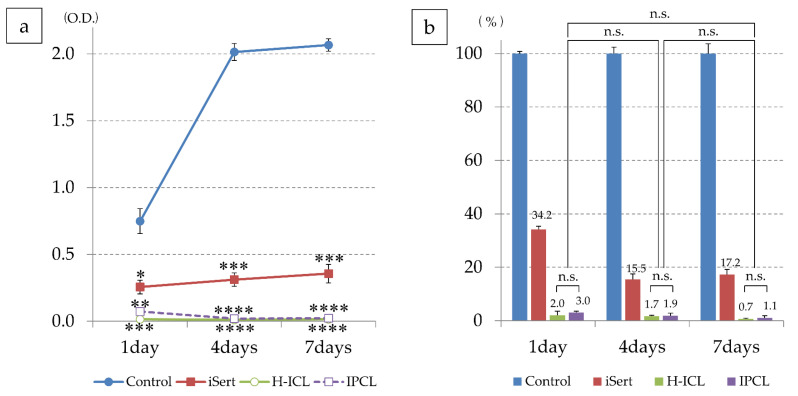
Proliferation of iHLEC-NY2 cells measured by WST-8 assay: (**a**) Measurement results based on absorbance values compared to those on a multi-well cell culture plate. * *p* < 0.05, ** *p* < 0.01, *** *p* < 0.005, **** *p* < 0.001; (**b**) Comparison results when cell viability in the control group was set as 100%. There was no significant difference in cell viability between H-ICL and IPCL. No significant difference was observed with respect to the culture period, but there was a slight tendency of decreased cell viability compared to the control group. n.s.: no significant difference.

On day 1 of culture, light absorbance at wavelength 450 nm 3 h after addition of WST-8 reagent was significantly lower in IOL (*p* < 0.05), H-ICL (*p* < 0.005), and IPCL (*p* < 0.01) compared to that by iHLEC-NY2 cells seeded on the control multi-well plate. On day 4 of culture, although iHLEC-NY2 cells showed proliferation in the control group, while cells were proliferating in the IOL, absorbance was significantly lower (*p* < 0.005), and in the H-ICL and IPCL even more so (*p* < 0.001) than in the control group. On day 7, the control group had nearly reached confluence, resulting in a plateau in the increase in absorbance. Although the IOL showed increased proliferation compared to day 4, its absorbance was significantly lower compared to control (*p* < 0.005), and both H-ICL and IPCL showed even lower absorbance (*p* < 0.001, Figure 10a).

IOL appeared to have lower cell viability on day 4 than day 1, but this is because absorbance on day 4 was much higher than that on day 1 in the control. There was no difference in iHLEC-NY2 cell activity between H-ICL and IPCL on days 1, 4, and 7 of culture, and cell activity of H-ICLs and IPCLs tended to decrease as the number of culture days increased (Figure 10b).

## 4. Discussion

In this study, compression load and static Young’s modulus were measured to evaluate lens flexion resistance (stiffness) in two types of lens: H-ICLs and IPCLs. In addition, adhesion of cells to H-ICLs and IPCLs made of different materials was investigated. In compression load evaluation, lenses were investigated under each of the two conditions, optic section restrained and unrestrained. The latter condition simulated the state where the haptic supports of the phakic-ICL are flexed to tuck behind the iris after intraocular insertion. Since this is performed one side at a time when fixing in the eye, flexion of 2.25 mm is the flexion amount required at that stage. The compression load to the lens hardly differed by size of H-ICL. IPCL varieties −13.50 D and −8.00 D showed a compression load similar to that of H-ICL. When comparing the two types of lenses, increasing flexion to 4.5 mm tended to require higher load for H-ICLs than IPCLs, but the difference was not significant.

In contrast, by restraining the center of the optic section, its free movement is restricted, allowing evaluation of response of the haptic supports to compression load. The first area to be affected by increased load was around the junction of the lens optic section and haptic support. For both types of lenses, at flexion of approximately 1 mm, the junction between the optic section and the haptic support starts to bend, H-ICL lengths 13.2 mm and 12.1 mm required about the same load as IPCL power −13.50, and H-ICL length 12.6 mm and IPCL power −8.00 D required about the same load. When comparing the two types of lenses, increasing flexion required higher load for H-ICL. Based on these results, it can be inferred that in the case of compression load to phakic-ICLs from the lens side through flexion of the haptic support to tuck it behind the iris after intraocular insertion, the junction between the lens optic section and haptic support plays a central role, generating reactive bending of the lens in response to the applied load.

**Figure 11:**
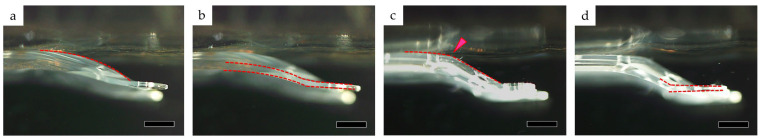
Comparison of the side profiles of H-ICLs and IPCLs: (**a**) the junction from the optic section to the haptic support of H-ICL showed a gradual curved shape (red dotted line); (**b**) the thickness of the haptic support of H-ICL decreased towards the tip (red dotted line); (**c**) the junction from the optic section to the haptic support of IPCL showed a sharp shape transition (red dotted line) and slight convexity (red arrow); (**d**) the thickness of the haptic support of IPCL decreased towards the tip (red dotted line). The scale bar denotes 1 mm.

The H-ICL has a gradual curvature from the optic section to the optic/haptic junction. In contrast, the IPCL has a slightly more acute angle on the anterior side of the optic section compared to the H-ICL (Figure 11a,c). One of the reasons for this design of the IPCL is to maintain a greater distance between the IPCL and the crystalline lens, so they are less likely to touch each other.

We believe the difference in shape of the anterior surface of the optic section is related to the observation in the unrestrained optic experiment of slight convexity at the optic/haptic junction in the IPCL compared to H-ICL, which shows a gradual curve in the overall shape of the lens as flexion increases. This effect became more pronounced when the optic section was restrained, and the IPCL showed a similar convexity originating from the same region. In contrast, when flexion was below 0.8 mm to 0.9 mm, the anterior surface of the optic section of the H-ICL showed a gradual curve; however, once flexion exceeded 1.0 mm, bending of the haptic support was observed. It is presumed that this difference in lens shape is related to the difference in transverse compression load between H-ICL and IPCL.

Young’s modulus (longitudinal elasticity modulus) indicates the property of a material to return to its original shape when an applied external force is removed. Static Young’s modulus was calculated from the displacement of H-ICL or IPCL before and after applied load transferred to the center of the optic section of the lens. Displacement relative to the surface was significantly greater in IPCL. The relationship between compression load, Young’s modulus, and displacement (shrinkage) is expressed in the following formula.

δ = PL/EA

δ: displacement; P: load (compression load); L: length of material; E: Young’s modulus; A: cross-sectional area.

Young’s modulus represents the degree of change (strain: ε) that occurs in an object when it undergoes deformation compared to its original state. It is determined by comparing the resistance force (stress: σ) per unit area exerted on the material in the opposite direction when an external force is applied. Young’s modulus can be mathematically quantified as E = σ/ε. Higher values of Young’s modulus indicate that the material is more rigid or has a greater stiffness.

Based on the results of compression load and static Young’s modulus measurements in this study, it was observed that H-ICL exhibited a stronger reaction to the transverse load applied to the lens compared to IPCL. Conversely, IPCL showed a significantly stronger reaction to the force that was transferred to the surface of the center of the optic section. These findings are believed to be associated with the lens shape, particularly the continuous design from the optic section to the haptic support. However, when the optic section was unrestrained as during lens insertion behind the iris, both H-ICL and IPCL required similar compression loads to achieve flexion of 2.25 mm, except for the −5.00 D IPCL. The lower load for −5.00 D IPCL may be attributed to factors such as lens power and slight variation in optic section thickness, although detailed information on the lens design was not available. Furthermore, experiments with the optic section restrained revealed the optic/haptic junction tended to bend more easily for flexion up to approximately 1 mm, which may give the impression of lens softness to an ophthalmologist performing implant surgery. During surgery, both H-ICL and IPCL are inserted into the anterior chamber of the eye using an injector. In our experience, ophthalmologists’ impressions were that unfolding the lenses after ejection from the injector their stiffness appeared similar. It is worth noting that while Young’s modulus indicates that IPCL is stiffer than H-ICL in the optic section, as verified by the load on the surface of its center, the haptic support is the one manipulated intraocularly during surgery. Consequently, it may be challenging for ophthalmologists to perceive IPCL as being stiffer. In the present study, lenses of different sizes and diopters were used, and the results differed among these variables. However, as the size and power of a lens is selected to fit the size and needs of an eye in clinical practice, we considered that these differences have an insignificant impact in clinical practice. Furthermore, since the lenses used in the study were minus lenses (which correct nearsightedness), the influence of difference in diopter power was considered to be minimal to insignificant.

When the surface morphology of H-ICL and IPCL lenses was analyzed using atomic force microscopy, it was reported that the surface of IPCL was significantly smoother than that of H-ICL [28]. In the present study, we investigated cell adhesion to H-ICL and IPCL lenses, which are made of different materials. When compared to control cell culture dishes and lens control IOL, we observed cell adhesion on day 1 of culture. However, the cells subsequently detached, and there was minimal cell adhesion to either H-ICL or IPCL. Although cell adhesion is influenced by the ‘roughness’ of the texture of the lens surface, the surface roughness of H-ICL and IPCL was not significant enough for cell adhesion, and thus it seems likely that the surfaces of H-ICL and IPCL were sufficiently smooth to hinder cell adhesion.

In our previous study involving 10 patients and 13 eyes, the mean ± SD duration of H-ICLs in the posterior chamber (from implantation to removal) was 10.5 ± 2.7 years. Upon extraction, there was no opacification or coloration of the removed H-ICL and its light transmittance was nearly equivalent to that of unused lenses. The lens surfaces were examined using scanning electron microscopy, and no obvious deposits were observed, indicating long-term in vivo biocompatibility and stability of the H-ICL [29].

Of the two lenses studied here, H-ICLs are made of a hydrophilic material called collamer, a biocompatible material that is a mixture of polymer and collagen designed to reduce lens weight, improve hydrophilicity, and facilitate gas and nutrient exchange [15]. Collamer is comprised of 60% HEMA, 36% water, 3.8% benzophenone, and 0.2% porcine collagen [30]. Initially, there was no hole in the center of the lens, but a lens with a 0.36 mm hole (KS-AquaPORT^®®^, STAAR Surgical) was introduced to provide a circulatory pathway for aqueous humor. With the advent of this type, the need for peripheral iridotomy was eliminated [30,31]. Furthermore, the diameter of the lens optic increased to 6.1 mm, which reduced the incidence of postoperative halo glare. Postoperative VA was reported improved in a large patient cohort study [6], as was postoperative uncorrected VA, postoperative refractive error, and stability [20,29]. Safety and VA after such lens removal has also been reported [32]. IPCL, in contrast, uses a hybrid hydrophilic acrylic that can maintain long-term visual function in the eye. The material of IPCL is a hybrid material with a high water content that prevents proteins and other contaminants from adhering to the lens. The diameter of the lens optic is large (6.6 mm), and the holes are trapezoidal rather than cylindrical to suppress halo glare. There are seven holes in the optic section, which not only allow aqueous humor circulation, but contribute to a lower incidence rate of cataracts and glaucoma [33]. However, as yet, there are no reports of long-term follow-up of IPCLs, as is the case for H-ICLs [34]. The phakic-ICL, which uses the same hybrid hydrophilic acrylic material as the IPCL, is an Eyecryl lens made by WEYEZER of Switzerland, although it was not used in this study. The performance of the lens is the same as that of the H-ICL lens, but the diameter of the lens optic is smaller at 5 mm, which has the disadvantage that halo glare is more likely to occur than in the H-ICL and IPCL.

A limitation of this study is that not all types of H-ICL and IPCL were investigated. The reason for the lower compression load observed in the −5.00 D IPCL in the present study is unknown, as detailed information regarding the lens design has not been disclosed by the manufacturer. Since new lenses were used, any effects of lens aging are unknown. In addition, the current experiment was conducted in BSS Plus solution buffer and no viscoelastic material was used. The cell adhesion experiments were conducted using immortalized lens epithelial cells, and it should be noted that the study did not verify the adhesion of cells with strong adhesive properties, such as macrophages, which may infiltrate tissues in case of intraocular inflammation.

## 5. Conclusions

In the present study, we compared the physical properties of H-ICL and IPCL, which have been used in a relatively large number of case reports. The required transverse compression load for flexion tended to be greater for the H-ICL, and Young’s modulus was greater for the IPCL with respect to the force exerted to the center of anterior surface of the optic section. These observations suggest that in the IPCL, the joint between the lens optic section and the haptic support, designed to maintain distance from the crystalline lens, mitigates the effects of transverse compression load. Since there are few reports on the long-term clinical outcomes of IPCL, it is unclear whether or to what extent the shape and position of an implanted IPCL may change with time caused by contact with the posterior surface of the iris or for reasons unknown. Therefore, long-term observation of patients implanted with IPCL is necessary to fully understand their clinical outcomes.

## Figures and Tables

**Table 1:** Compression load (mean ± SD) by flexion for H-ICL- and IPCL-type lenses.

Flexion (mm)	H-ICL (mN)	IPCL (mN)
0.50	0.06 ± 0.12	−0.04 ± 0.16
1.00	0.14 ± 0.10	0.09 ± 0.07
1.50	0.19 ± 0.08	0.11 ± 0.14
2.00	0.25 ± 0.09	0.18 ± 0.13
2.25	0.30 ± 0.09	0.21 ± 0.20
2.50	0.38 ± 0.10	0.24 ± 0.15
3.00	0.51 ± 0.16	0.34 ± 0.23
3.50	0.70 ± 0.20	0.38 ± 0.19
4.00	0.87 ± 0.24	0.41 ± 0.21
4.50	1.03 ± 0.26	0.53 ± 0.26

**Table 2:** Compression load (mean ± SD) by flexion for H-ICL- and IPCL-type lenses.

Flexion (mm)	H-ICL (mN)	IPCL (mN)
0.50	1.24 ± 0.44	0.97 ± 0.71
1.00	2.05 ± 0.59	1.41 ± 0.90
1.50	1.79 ± 0.36	0.90 ± 0.81
2.00	3.84 ± 1.30	0.88 ± 1.29
2.25	4.91 ± 1.88	1.22 ± 1.81

**Table 3:** Absorbance values (mean ± SD).

Culture Day	Control (O.D.)	IOL (O.D.)	H-ICL (O.D.)	IPCL (O.D.)
Day-1	0.748 ± 0.093	0.256 ± 0.051	0.015 ± 0.006	0.073 ± 0.015
Day-4	2.014 ± 0.064	0.311 ± 0.051	0.011 ± 0.003	0.020 ± 0.009
Day-7	2.066 ± 0.046	0.356 ± 0.069	0.015 ±0.002	0.023 ± 0.008

O.D., optical density.

## Data Availability

Data sharing not applicable.

## Supplementary Materials

The following supporting information can be downloaded at: https://www.mdpi.com/article/10.3390/medicina59071282/s1, Video S1: Compression load of H-ICL with optic section restrained, Video S2: Compression load of IPCL with optic section restrained.
